# Caveolae provide a specialized membrane environment for respiratory syncytial virus assembly

**DOI:** 10.1242/jcs.198853

**Published:** 2017-03-15

**Authors:** Alexander Ludwig, Tra Huong Nguyen, Daniel Leong, Laxmi Iyer Ravi, Boon Huan Tan, Sara Sandin, Richard J. Sugrue

**Affiliations:** 1 School of Biological Sciences, Nanyang Technological University, 60 Nanyang Drive, Singapore637551; 2 Detection and Diagnostics Laboratory, DSO National Laboratories, 27 Medical Drive, Singapore117510

**Keywords:** Caveolae, Caveolin, Cavin, Respiratory syncytial virus, Virus assembly, Virus envelope

## Abstract

Respiratory syncytial virus (RSV) is an enveloped virus that assembles into filamentous virus particles on the surface of infected cells. Morphogenesis of RSV is dependent upon cholesterol-rich (lipid raft) membrane microdomains, but the specific role of individual raft molecules in RSV assembly is not well defined. Here, we show that RSV morphogenesis occurs within caveolar membranes and that both caveolin-1 and cavin-1 (also known as PTRF), the two major structural and functional components of caveolae, are actively recruited to and incorporated into the RSV envelope. The recruitment of caveolae occurred just prior to the initiation of RSV filament assembly, and was dependent upon an intact actin network as well as a direct physical interaction between caveolin-1 and the viral G protein. Moreover, cavin-1 protein levels were significantly increased in RSV-infected cells, leading to a virus-induced change in the stoichiometry and biophysical properties of the caveolar coat complex. Our data indicate that RSV exploits caveolae for its assembly, and we propose that the incorporation of caveolae into the virus contributes to defining the biological properties of the RSV envelope.

## Abstract (precis)

**Highlighted Article:** The assembly of respiratory syncytial virus occurs in caveolar membranes and caveolar coat components are incorporated into the viral envelope.

## INTRODUCTION

Respiratory syncytial virus (RSV) is a leading cause of lower respiratory tract infection in young children. The assembly of RSV takes place on the surface of infected cells, where the viral ribonucleoproteins (RNPs) are packaged into filamentous particles that are bounded by a viral envelope (Bachi and Howe, 1973; Bachi, 1988; Santangelo and Bao, 2007; Roberts et al., 1995). The viral envelope is derived from the plasma membrane of the host cell and contains integral viral membrane proteins such as the fusion (F) protein and the attachment glycoprotein (G). The G protein interacts with the matrix (M) protein and thus connects the viral RNPs to the viral membrane (Ghildyal et al., 2005; Henderson et al., 2002; Liljeroos et al., 2013). Although many molecular aspects of the virus assembly process have been defined (Shaikh and Crowe, 2013), the processes underlying the formation of the RSV envelope are poorly understood.

Cholesterol-rich microdomains (or lipid rafts) are critical for the morphogenesis and budding of several enveloped viruses, as well as for many post-assembly aspects of the virus infectious cycle including attachment, entry, uncoating, protein transport and sorting (Nayak and Hui, 2004). Lipid rafts are also required for RSV morphogenesis, as procedures that disrupt such microdomains inhibit RSV particle assembly and transmission (Yeo et al., 2009; Chang et al., 2012; Brown et al., 2004, 2002a; McCurdy and Graham, 2003). In addition, various raft-associated proteins are associated with RSV particles (Brown et al., 2002a,b, 2004; Jeffree et al., 2003). This suggests that lipid raft microdomains provide cellular platforms for RSV assembly, and that the incorporation of raft components into the virus may impart important biological properties to the viral envelope. However, much of the evidence supporting a role of lipid rafts in RSV assembly is based on non-selective perturbations and assays, such as the extraction of viral proteins in non-ionic detergents at 4°C, and the use of generic raft markers and cholesterol depletion. As a consequence, the specific contribution and functional significance of individual lipid microdomains in RSV morphogenesis is largely undefined. Furthermore, it is unclear whether raft-associated components are actively recruited to RSV particles or if they are passively incorporated during the virus assembly process in lipid rafts.

Caveolae are bulb-shaped invaginations in the plasma membrane that are rich in cholesterol and sphingolipids (Stan, 2005). They are a unique type of lipid raft and their characteristic morphology and protein composition discriminates them from other raft- and non-raft membranes. Caveolins (caveolin-1, -2, and -3) and cavins (cavin 1–4, also known as PTRF, SDPR, PRKCDBP/SRBC and MURC, respectively) are the key structural and functional components of caveolae (Hansen and Nichols, 2010; Kovtun et al., 2015). Together, they assemble into a large 80S protein complex that constitutes the protein coat of caveolar membranes (Ludwig et al., 2013, 2016; Shvets et al., 2014). Whereas caveolins are integral membrane proteins that are inserted into the inner leaflet of the lipid bilayer (Ariotti et al., 2015), cavins are cytoplasmic proteins that oligomerize into large complexes (Bastiani et al., 2009; Hayer et al., 2010a; Ludwig et al., 2013) and bind to membranes rich in phosphatidylserine (PS) and phosphatidylinositol 4,5-bisphosphate [PI(4,5)P_2_] (Kovtun et al., 2015, 2014). Caveolin-1 and cavin-1 are essential for the formation of caveolae *in vivo* (Drab et al., 2001; Hill et al., 2008; Liu and Pilch, 2008), whereas caveolin-2 (Razani et al., 2002) and cavins 2–4 (Hansen et al., 2013) are dispensable.

Caveolae have been implicated in various cellular processes including lipid metabolism and trafficking, endocytosis and signaling (Cheng and Nichols, 2016; Parton and del Pozo, 2013). We and others have previously demonstrated an association of caveolin-1 with RSV filaments in virus-infected cells (Brown et al., 2002a; Kipper et al., 2015; Radhakrishnan et al., 2010). Moreover, a role for caveolin-1 in the morphogenesis of other enveloped viruses, including influenza virus (Sun et al., 2010), dengue virus (García Cordero et al., 2014) and parainfluenza virus 5 (PIV-5) (Ravid et al., 2010), has been described. Although the combined data suggest a function of caveolae in viral biogenesis, an association with caveolin-1 alone does not directly demonstrate the involvement of caveolae in virus morphogenesis. In addition, siRNA-mediated knockdown of caveolin-1 was shown to have no effect on RSV morphogenesis and infection in cultured cells (Kipper et al., 2015), and there is some evidence that caveolin-1 might have an anti-viral role during virus infection (Gabor et al., 2013; Bohm et al., 2014; He et al., 2016). Thus, the role of caveolin-1 and caveolae in virus-infected cells remains unclear.

In this study, we have employed a combination of light and electron microscopy, biochemistry, live-cell imaging, and RNAi to examine the localization, biochemical properties, dynamics and functions of caveolae in the context of RSV filament assembly. Our data show that RSV assembly occurs within caveolae and that caveolae are actively recruited to and incorporated into the RSV envelope. To our knowledge, this is the first detailed study to address the biology of a specific lipid microdomain during RSV assembly.

## RESULTS

### Caveolin-1 and cavin-1 are associated with RSV filaments

**Fig. 1.**
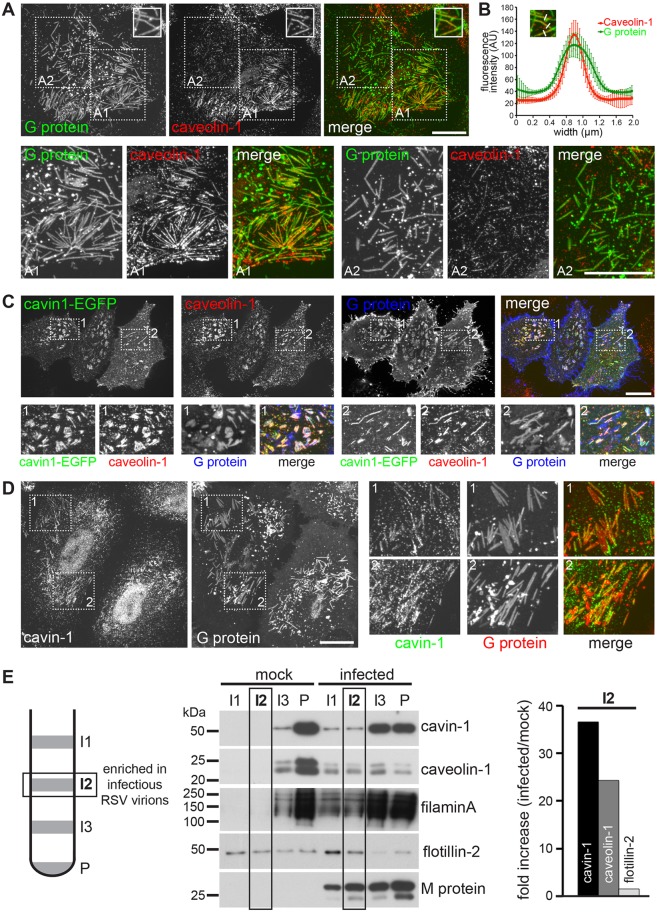
**Caveolin-1 and cavin-1 are associated with RSV filaments.** (A) Confocal micrographs of RSV-infected HeLa cells (22 hpi) stained with antibodies against caveolin-1 and RSV G protein. A1 and A2, close-up of boxed regions in A. (B) Average fluorescence intensity distribution of caveolin-1 and G protein in viral filaments (*n*=10 line scans with a representative image shown in the inset, error bars are standard deviations). (C) Confocal micrographs of RSV-infected HeLa cells (22 hpi) stably transfected with cavin-1–EGFP and co-stained with antibodies against caveolin-1 and RSV G protein. (D) Indirect immunofluorescence and confocal microscopy of RSV-infected HeLa cells (22 hpi) using antibodies against cavin-1 and RSV G protein. (E) Isolation of RSV virions from HEp-2 cells. A schematic of the sucrose gradient is shown. Three interphase fractions (I1–I3) and the pellet (P) were subjected to immunoblotting using the indicated antibodies. The relative enrichment of caveolin-1, cavin-1 and flotillin-2 in I2 was determined by densitometry and plotted (representative of two independent experiments). Scale bars: 20 µm.

To study the distribution of caveolar proteins in virus-infected cells, HeLa cells were infected with RSV and processed for indirect immunofluorescence at 20–24 h post infection (hpi). Endogenous caveolin-1 and the viral G protein colocalized in RSV filaments as assessed by confocal microscopy (Fig. 1A,B), confirming previous observations (Brown et al., 2002a; Kipper et al., 2015). No filamentous staining was observed for caveolin-1 in mock-infected HeLa cells (Fig. S1A), indicating a virus-induced change in caveolin-1 distribution. The degree to which caveolin-1 and the viral G protein colocalized was somewhat variable. Whereas many filaments were strongly stained by the anti-caveolin-1 antibody (Fig. 1A1), others were stained only faintly (Fig. 1A2). To examine the specificity of the caveolin-1 association with RSV, the distribution of the raft marker flotillin-2 was examined (Glebov et al., 2006; Frick et al., 2007). Although flotillin-2 colocalized with the viral F protein in perinuclear late endosomes and lysosomes, confocal imaging revealed no evidence for an association of flotillin-2 with RSV filaments (Fig. S1B–D). This indicates a selective association of caveolin-1 with RSV.

Cavin-1 forms a stable protein complex with caveolin-1 (Ludwig et al., 2013), prompting us to test whether cavin-1 was also recruited to RSV filaments. This was first addressed in a HeLa cell line stably transfected with cavin-1–EGFP (Ludwig et al., 2013). As expected, in mock-infected cells cavin-1–EGFP and caveolin-1 colocalized in a punctate pattern in the membrane (Fig. S1A); no filamentous staining pattern was observed under these experimental conditions. In contrast, in RSV-infected cells cavin-1–EGFP, caveolin-1 and the viral G protein all colocalized within RSV filaments (Fig. 1D). Staining of RSV-infected HeLa cells with specific anti-cavin-1 antibodies confirmed the association of cavin-1 with the viral filaments (Fig. 1D). The degree of colocalization between caveolin-1 and cavin-1–EGFP was identical in mock-infected cells (Pearson coefficient: 0.78±0.07; mean±s.d.; *n*=16 cells) and RSV-infected cells (Pearson coefficient: 0.77±0.07; mean±s.d.; *n*=19 cells). Colocalization between cavin-1–EGFP (or caveolin-1) and the viral G protein was again more variable, but individual filaments or groups of filaments exhibited a very high degree of colocalization (Pearson coefficient: 0.72±0.1; mean±s.d.; *n*=17 areas in ten cells). This provides strong evidence that both caveolin-1 and cavin-1 are associated with RSV filaments.

To support the above observation, we isolated RSV virions from cultured HEp-2 cells by sucrose density centrifugation (Radhakrishnan et al., 2010), and examined the cell-free virus particles for the presence of cavin-1 and caveolin-1. Three interphase (I1, I2 and I3) fractions and the pellet (P) fraction were collected from the gradient (Fig. 1E). I2 is enriched in infectious RSV particles, whereas I1 and I3 contain viral antigen that is associated with non-infectious viral material (Radhakrishnan et al., 2010). Immunoblotting and densitometry showed that caveolin-1 and cavin-1 become highly enriched in the I2 interphase in virus-infected cells (Fig. 1E). A similar enrichment in the I2 interphase was observed for filamin-A, an actin-binding protein that is associated with virus filaments (Radhakrishnan et al., 2010; Shaikh et al., 2012) and has been suggested to interact with caveolin-1 (Stahlhut and van Deurs, 2000). In contrast, the relative abundance of flotillin-2 in the I2 interphase was largely unchanged compared to control gradients. We conclude that caveolin-1 and cavin-1, the two key structural and functional components of caveolae, are incorporated into RSV particles.

### The caveolar protein coat is incorporated into the RSV envelope

Next, we examined the spatial relationship between caveolae and RSV filaments by transmission electron microscopy (TEM) of cells expressing a caveolin-1–APEX2–EGFP fusion protein. APEX2 is an engineered ascorbate peroxidase that serves as a genetically encoded tag for the selective staining of proteins by EM (Lam et al., 2015). In line with our previous data (Ludwig et al., 2016), expression of caveolin-1–APEX2–EGFP in mock-infected HeLa cells resulted in the specific staining of caveolar membranes (Fig. S2).

**Fig. 2.**
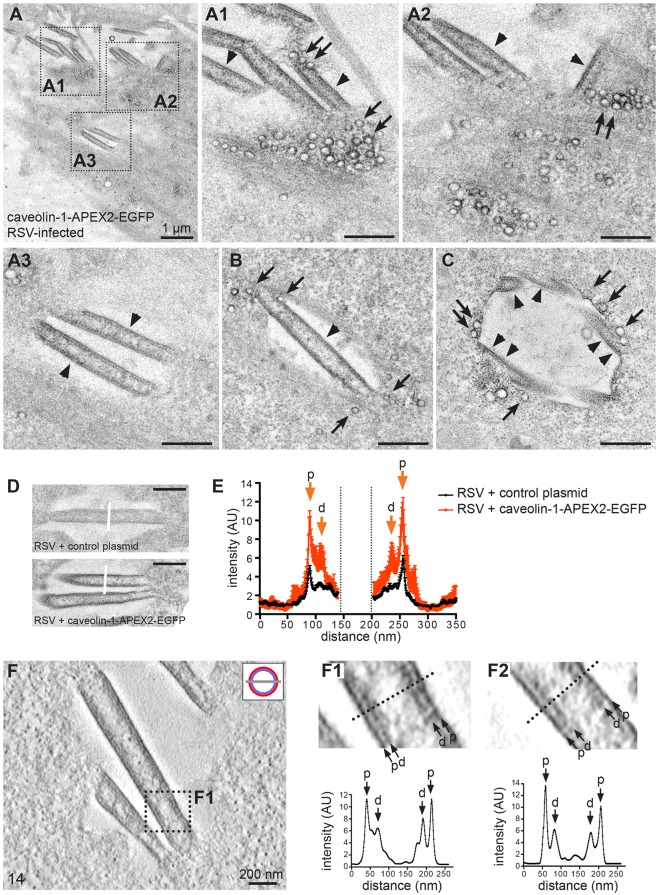
**Caveolin-1 is incorporated into the RSV envelope.** (A–C) Representative transmission electron micrographs of RSV-infected HeLa cells (22 hpi) transfected with caveolin-1–APEX2–EGFP. (A1–A3) Close-ups of regions boxed in A. Note the electron-dense stain on caveolar membranes (arrows) and the RSV envelope (arrowheads), and the presence of caveolae at the base of virus filaments (A1,A2,B,C). (D) Representative micrographs of control RSV filaments (osmium post-fixation alone, top) and RSV filaments stained with caveolin-1–APEX2–EGFP (bottom). (E) Quantification of staining intensity across the filament width (as indicated in D) in control (black line) and caveolin-1–APEX2–EGFP-stained RSV filaments (red line). Shown are the averages and standard deviations of 22 line scans each (*n*=6 cells). Note the significant contrast increase in the RSV envelope in caveolin-1–APEX2–EGFP-expressing cells, and the presence of two discrete peaks (p, membrane-proximal; d, membrane-distal). (F) Representative tomographic slice through an RSV filament stained with caveolin-1–APEX2–EGFP. The cartoon depicts the slice position (red, viral envelope; light blue, viral matrix). F1 and F2 are representative close-up views; images were contrast-enhanced and gaussian-filtered. Line scans across the filament are shown. Scale bars are 500 nm unless otherwise stated.

RSV-infected HeLa cells expressing caveolin-1–APEX2–EGFP were processed for APEX2 staining at 20 hpi and imaged by TEM. Previous EM work has shown that RSV filaments can be identified as projections that are ∼150 nm wide and several micrometers long on the surface of infected cells (Bachi and Howe, 1973; Brown et al., 2002a; Roberts et al., 1995). Viral filaments were readily observed in our caveolin-1–APEX2–EGFP-stained and RSV-infected samples (Fig. 2A–C). Importantly, the viral membrane was strongly stained, both compared to the surrounding cellular membrane and compared to the filament shaft (Fig. 2A1–A3,B). In addition, we often observed single caveolae and clusters of caveolae at or very close to the filament base (Fig. 2A1,A2,B,C). In some cases, caveolae appeared to be directly attached to the viral membrane (Fig. 2A1,B). This indicates that caveolae are recruited to sites of RSV assembly, and that caveolin-1 (and cavin-1, as inferred from our light microscopy data) is incorporated into the RSV envelope, possibly via fusion of caveolae with the viral membrane.

**Fig. 3.**
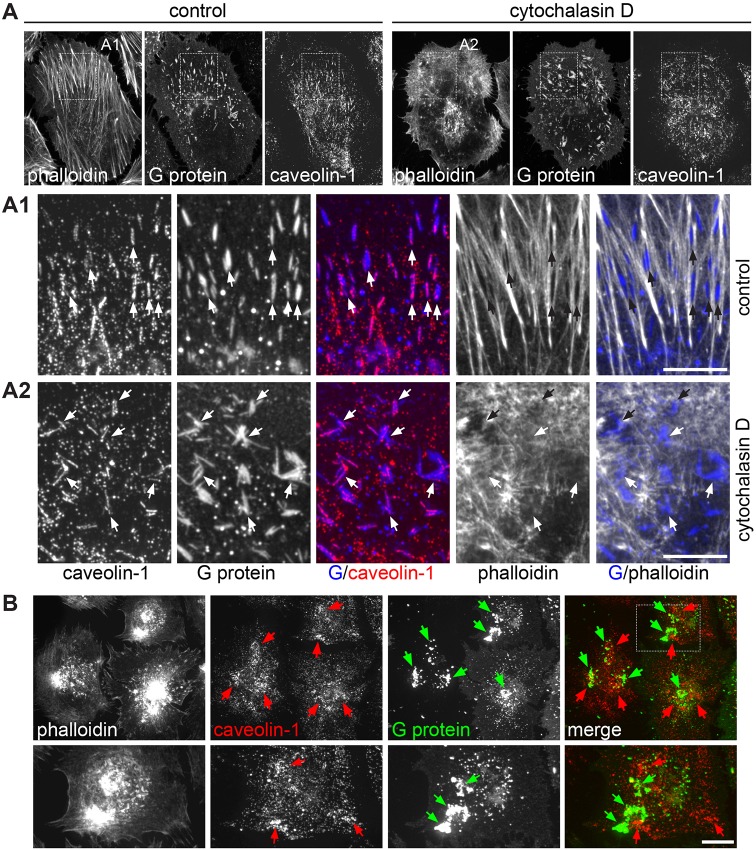
**Caveolin-1 is recruited to RSV filaments in an actin-dependent manner.** (A,B) RSV-infected HeLa cells were treated at 14 hpi with 500 nM cytochalasin D (A2,B) or left untreated (A1). Cells were fixed at 20 hpi and stained with antibodies against caveolin-1 and G protein, and phalloidin–FITC. Confocal micrographs are shown. (A1) RSV filaments are aligned along actin fibers. (A2) Partial disruption of the actin network with cytochalasin D causes distortion and aggregation of RSV filaments. Arrows indicate virus filaments (B) RSV-infected and cytochalasin-treated HeLa cells showing complete disruption of the actin cytoskeleton. Note the lack of RSV filaments and the loss of colocalization between caveolin-1 (red arrows) and G protein (green arrows). Representative data of two independent experiments are shown. Scale bars: 10 µm.

We compared the membrane contrast in RSV filaments in cells transfected with caveolin-1–APEX2–EGFP with that of cells expressing a control plasmid (Fig. 2D; Fig. S3A). Quantification of this data showed a strong and significant increase in staining intensity in the viral membrane in caveolin-1–APEX2–EGFP transfected samples compared to control samples (Fig. 2E). This analysis confirmed that the electron-dense stain in the viral membrane was due to APEX2-mediated deposition of a DAB-osmium polymer and not a result of general membrane contrast produced by osmium alone. Interestingly, staining of caveolin-1 generated electron density at two locations within the viral particle. One electron-dense layer appeared to be on the viral membrane (membrane proximal layer) and was more intense than a similar layer in control cells. A second unique electron-dense layer that was located 15–20 nm beneath the viral membrane (membrane distal layer) was also noted (Fig. 2E). In contrast, the staining produced on caveolar membranes was restricted to a single layer of electron density (Fig. S2C). 3D electron tomography of caveolin-1–APEX2–EGFP-stained RSV filaments (Fig. 3F; Movie 1) confirmed the dual distribution of caveolin-1 in the viral envelope (Fig. 3F1,F2). Importantly, electron tomography of RSV filaments stained ‘conventionally’ (with uranyl acetate and lead citrate) revealed a different staining pattern (Fig. S3B; Movie 2). Uranium and lead efficiently stained viral RNPs within the filament shaft, as expected. Moreover, and in striking contrast to filaments stained for caveolin-1–APEX2–EGFP, no major increase in electron density was observed in the viral envelope in uranium- and lead-stained specimen. Taken together, our EM analyses indicate that incorporation of caveolin-1 and cavin-1 into the RSV envelope generate a protein coat that is distinct from that on caveolar membranes.

### Caveolin-1 is recruited to RSV in an actin-dependent manner

Since caveolae are aligned along actin stress fibers (Echarri and Del Pozo, 2015) (Fig. S4A), and RSV morphogenesis and infection is actin-dependent (Jeffree et al., 2007; Ravi et al., 2013; Burke et al., 1998; Ulloa et al., 1998), we asked whether actin was required for the recruitment of caveolae to the virus. To determine the time point at which caveolae become associated with RSV, the distribution of caveolin-1 and the viral G protein was studied in fixed RSV-infected HeLa cells at between 12 and 18 hpi (Fig. S4B–D). At 12–14 hpi, the G protein formed relatively large punctate structures in the cell membrane, and no significant colocalization with caveolin-1 was detected at these early time points of infection. At 16–18 hpi, caveolin-1 and the G protein colocalized in punctate structures, in short and slightly elongated structures, and in longer filamentous structures. This suggests that caveolin-1 is recruited to sites of RSV assembly just prior to the onset of filament formation. To analyze the involvement of actin in this process, RSV-infected HeLa cells were treated at 14 hpi with moderate concentrations (500 nM) (Wakatsuki et al., 2001) of the actin-depolymerizing drug cytochalasin D and examined by confocal microscopy at 20 hpi. In non-treated HeLa cells, many RSV filaments were aligned along actin fibers (Fig. 3A1), consistent with previous observations (Jeffree et al., 2007; Ravi et al., 2013; Burke et al., 1998; Ulloa et al., 1998). Cytochalasin D treatment resulted in a mixed population of cells showing partial (Fig. 3A2) to full disruption (Fig. 3B) of the actin network. Partial disruption of actin stress fibers caused aggregation and distortion of RSV filaments, but had no or little effect on the association of caveolin-1 with the virus (Fig. 3A2). In cells that had lost all actin stress fibers, caveolin-1 and the G protein formed large aggregates in close proximity to the remaining F-actin (Fig. 3B). Under these conditions, RSV filament formation was inhibited, and colocalization between caveolin-1 and the G protein no longer evident. We conclude that actin is required for RSV morphogenesis and for the recruitment of caveolin-1 to the viral G protein.

### RSV filament assembly occurs within caveolar membranes

**Fig. 4.**
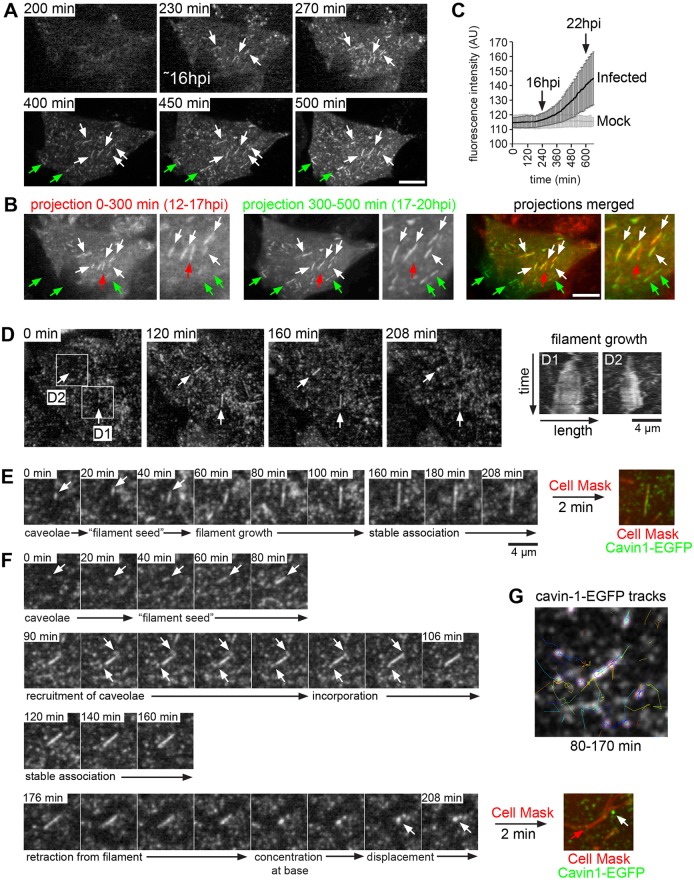
**RSV filament assembly occurs within caveolar membranes.** (A) HeLa cells stably transfected with cavin-1–EGFP were infected with RSV and imaged live at between 12 and 22 hpi by spinning disk microscopy using a frame rate of 10 min. Still images at the indicated time points are shown; contrast was enhanced for images in the top row. Note the appearance of filamentous structures at 230 min (∼16 hpi). (B) Two average intensity projections (0–300 min and 300–500 min) of the time-lapse shown in A. White arrows indicate RSV filaments that persisted throughout the time-lapse, green arrows indicate *de novo* formation of filaments between 300 min and 500 min, and red arrows indicate the disappearance of a filament. Scale bars: 10 µm. (C) Quantification of cavin-1–EGFP fluorescence intensity in mock-infected and RSV-infected HeLa cells. Plotted are the mean fluorescence intensities and standard deviations for each time point (*n*=44 cells each). (D) HeLa cells stably transfected with cavin-1–EGFP were infected with RSV and imaged live at 17 hpi using a frame rate of 2 min. Two regions (D1 and D2) of *de novo* filament formation are boxed and shown as kymographs on the right, illustrating growth of the two filaments over time. (E,F) Time-lapse gallery of boxed regions in D (E is D1; F is D2). Following the 208 min time-lapse, cells were stained for 2 min with the fluorescent membrane dye CellMask Orange. (G) Automated tracking of cavin-1–EGFP puncta. Note that cavin-1–EGFP puncta are recruited to the filament ends.

To examine the recruitment of caveolae in live cells, HeLa cells expressing cavin-1–EGFP were infected with RSV and imaged at between 12 and 22 hpi by spinning disk microscopy. In agreement with our timecourse experiment in fixed cells (Fig. S4B–D), filamentous cavin-1–EGFP started to appear at ∼16 hpi, indicative of viral filament growth (Fig. 4A; Movie 3). Filament formation was most prominent at 16–18 hpi, but new filaments also formed at later time points (Fig. 4B, green arrows). RSV filaments labeled with cavin-1–EGFP exhibited an average length of 3.2±0.9 µm (mean±s.d.; *n*=79), but lengths of up to 6 µm were noted. The majority of these filaments were stable once formed (Fig. 4B, white arrows), although in some cases cavin-1–EGFP appeared to be lost from filaments over time (Fig. 4B, red arrows). Interestingly, we found that RSV morphogenesis coincided with a marked increase in cavin-1–EGFP fluorescence intensity (Fig. 4C). This apparent upregulation of cavin-1 protein was highly synchronized across infected cells within the culture, proceeded at an approximately linear rate until 22 hpi, and was not observed in mock-infected cells recorded under identical conditions. We conclude that caveolae are incorporated into nascent RSV filaments, and that this is accompanied by increased levels of cavin-1 protein (see below).

Next, we analyzed the incorporation of cavin-1 into RSV filaments with greater temporal resolution. We found that RSV filament growth originated from cavin-1–EGFP puncta (Fig. 4D–F; Movies 4–6). The puncta initially elongated into a short ‘filament seed’ (Fig. 4E,F), upon which additional cavin-1–EGFP puncta were recruited to and incorporated into the nascent viral particle, causing filament growth. Interestingly, automated fluorescence tracking revealed that cavin-1–EGFP was incorporated primarily at the filament ends (Fig. 4G; Movie 7). After filament growth had ceased, cavin-1–EGFP remained stably associated with most filaments for 1 h or longer (Fig. 4E). However, in some instances cavin-1–EGFP appeared to retract and then be displaced from the virus particle (Fig. 4F, 176–208 min). Staining of live cells with the fluorescent membrane dye CellMask confirmed that filaments that had lost cavin-1–EGFP were still present at the end of the recording (Fig. 4F). This suggests that the incorporation of cavin-1–EGFP, and thus caveolae, into the RSV envelope is a reversible process. Taken together, our data indicate that RSV morphogenesis is initiated within caveolar membranes, and that caveolae are subsequently recruited to and incorporated into the growing RSV filaments.

### RSV infection alters the stoichiometry of the caveolar coat complex by stabilizing cavin-1 protein

**Fig. 5.**
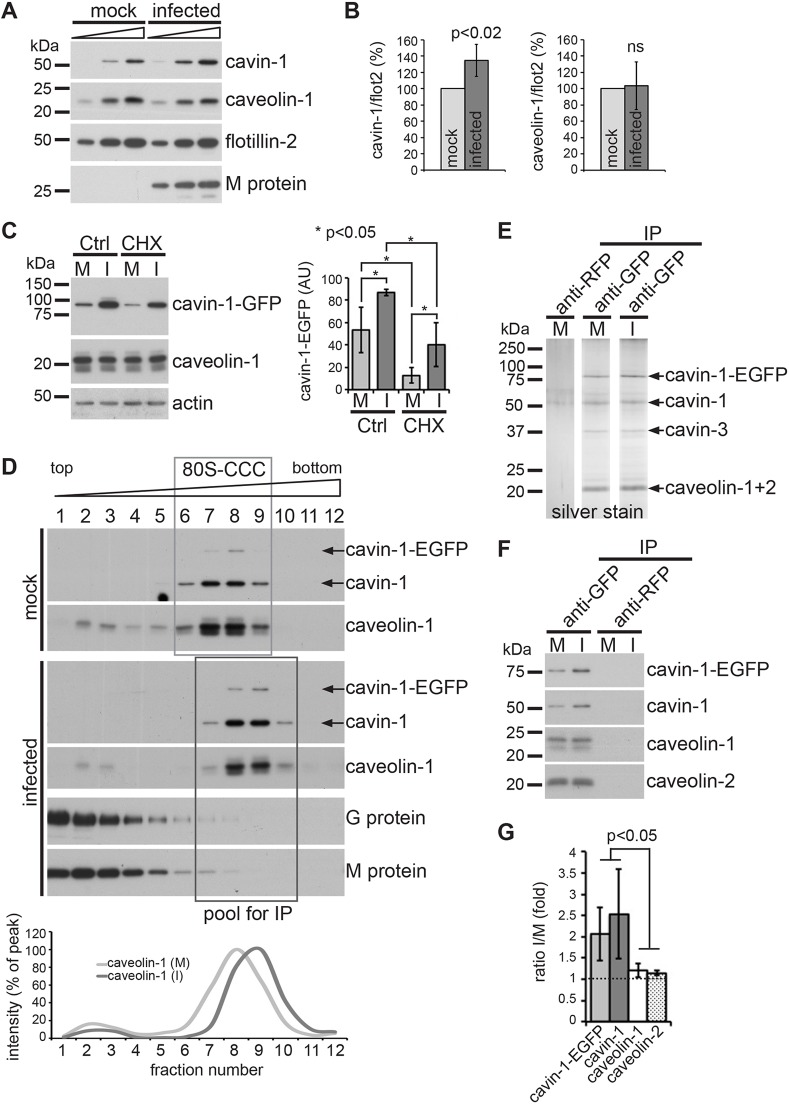
**RSV infection alters the stoichiometry of the caveolar coat complex by stabilizing cavin-1 protein.** (A) Immunoblotting of whole-cell lysates of mock-infected and RSV-infected HeLa cells (22 hpi) using the indicated antibodies. Increasing amounts (1×, 2×, 3×) of total cell lysates were loaded. (B) Quantification of the data shown in A (*n*=4; error bars indicate standard deviation) *P*<0.02; ns, not significant (Student's *t*-test). (C) HeLa cells stably expressing cavin-1–EGFP were treated at 16 hpi with 100 µg/ml cycloheximide (CHX), or were left untreated (Ctrl), and analyzed by immunoblotting at 22 hpi. Data was quantified by densitometry (*n*=3; error bars indicate standard deviation). *P<0.05 (Student's *t*-test). (D) Immunoblots of 10–40% sucrose gradient fractions prepared from mock-infected and RSV-infected HeLa cells stably expressing cavin-1–EGFP. Live cells were crosslinked at 22 hpi with 2 mM DSP. Cells were detergent extracted and the lysate fractionated. The 80S-CCC is boxed. The graph below shows the mean distribution of caveolin-1 in the gradients (*n*=3 separate experiments). (E) Silver stain gel of the affinity-purified 80S-CCC from mock-infected and RSV-infected cells. Immunoprecipitation was carried out from the boxed gradient fractions shown in D using anti-GFP or anti-RFP antibodies. (F) Immunoblotting of the affinity-purified 80S-CCC using the indicated antibodies. (G) Quantification of the data shown in F (*n*=3; error bars indicate standard deviation). *P*<0.05 (Student's *t*-test). M, mock infected; I, RSV infected.

We observed a marked upregulation of cavin-1 protein in RSV-infected cells between 16 hpi and 22 hpi (Fig. 4C). To study the significance of this finding, we quantified the relative cavin-1 and caveolin-1 protein levels in HeLa cell lysates prepared at 22 hpi from mock-infected and RSV-infected cells by immunoblotting (Fig. 5A). In line with our live-cell imaging data, we noted an ∼40% increase in endogenous cavin-1 protein in RSV-infected cells compared to mock-infected cells (Fig. 5B). In contrast, caveolin-1 and flotillin-2 protein levels were unchanged.

Global gene expression microarray analysis indicated that cavin-1 mRNA levels are unaltered in RSV-infected cells (Taye, B., Bioinformatics Institute, A*Star, Singapore; Chen, H., Genome Institute, A*Star, Singapore, and R.S., unpublished observations). To test whether the observed increase in cavin-1 protein expression was dependent upon *de novo* protein synthesis, mock-infected and virus-infected HeLa cells stably transfected with cavin-1–EGFP were treated at 16 hpi with cycloheximide, lysed at 22 hpi and examined by immunoblotting (Fig. 5C). In non-treated cells (no cycloheximide) RSV infection resulted in a significant increase in cavin-1–EGFP protein compared to mock-infected cells, as expected. Cycloheximide treatment significantly reduced cavin-1–EGFP protein levels, both in mock-infected and in RSV-infected cells, but had no effect on caveolin-1 expression. Interestingly, virus-infected cells treated with cycloheximide exhibited a significantly higher level of cavin-1–EGFP protein than was seen in mock-infected cells treated with cycloheximide. This suggests that RSV infection stabilizes cavin-1 protein via a post-translational mechanism, possibly by interfering with proteasome-mediated degradation of cavin-1 (Tillu et al., 2015).

Caveolins and cavins assemble into a large 80S protein complex (80S-CCC) that contains caveolin and cavin proteins at a defined stoichiometry (Ludwig et al., 2013, 2016). To examine whether the observed increase in cavin-1 protein in RSV-infected cells affected the assembly or stoichiometry of the 80S-CCC, we used an established *in situ* crosslinking assay. This assay is designed for optimal recovery of the caveolar protein coat by stabilizing interactions between cavin-1 (Ludwig et al., 2013, 2016). The crosslinked coat is then extracted from caveolar membranes by using stringent detergent and salt concentrations, and fractionated on 10–40% sucrose gradients. As expected, in mock-infected cells, caveolin-1, cavin-1 and cavin-1–EGFP formed a discrete peak in fractions 6–9 (Fig. 5D), which corresponds to the 80S-CCC (Ludwig et al., 2013). The 80S-CCC clearly formed in RSV-infected cells (fractions 7–10), but interestingly, the complex exhibited an increased buoyant density compared to that in mock-infected cells. Immunoprecipitation (IP) of the 80S-CCC using anti-GFP antibodies confirmed that cavin-1–EGFP, cavin-1, cavin-3, caveolin-1 and caveolin-2 formed a protein complex, both in mock-infected and in RSV-infected cells (Fig. 5E,F). Thus, interactions between caveolins and cavins, as well as the overall composition of the 80S-CCC, are maintained in RSV-infected cells. Interestingly, quantitative immunoblotting revealed a significant increase in cavin-1 and cavin-1–EGFP proteins in the 80S-CCC purified from infected cells compared to that purified from control cells (Fig. 5F,G), while caveolin-1 and caveolin-2 levels remained unchanged. We concluded that RSV infection alters the stoichiometry of the 80S-CCC by stabilizing cavin-1 protein during infection. Moreover, the combined data strongly suggest that the entire caveolar coat complex is incorporated into the RSV envelope.

### Caveolin-1 interacts with the RSV G and M protein complex

Next, we asked whether the incorporation of the caveolar coat into the RSV envelope is mediated by physical interactions between viral and caveolar proteins. The fully glycosylated 90 kDa RSV G protein is a prominent integral membrane protein in the RSV envelope that interacts with both the viral F and M proteins (Ghildyal et al., 2005; Jeffree et al., 2003; Low et al., 2008). The M protein, in turn, forms a protein complex with the M2-1 protein (Li et al., 2008). G and M proteins did not co-fractionate with the crosslinked and detergent-solubilized 80S-CCC in sucrose gradients (Fig. 5D) and were not associated with the affinity-purified 80S-CCC (data not shown). This suggests that viral envelope proteins were not crosslinked to components of the 80S-CCC, and that stringent detergent extraction may lead to their dissociation from the caveolar coat during the purification procedure.

**Fig. 6.**
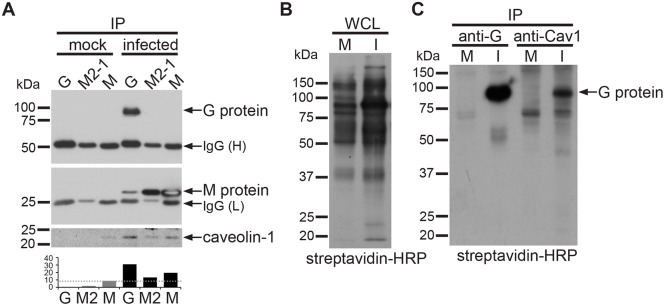
**Caveolin-1 interacts with the RSV G and M protein complex on the surface of infected cells.** (A) Immunoprecipitations of RSV G, M and M2-1 proteins from HeLa whole-cell lysates of mock-infected or RSV-infected cells (22 hpi). Immunoprecipitates were probed for G protein (top panel), M protein (middle panel) and caveolin-1 (bottom panel). Caveolin-1 protein was measured by densitometry (bottom graph). (B) Streptavidin–HRP blot of lysates of surface-biotinylated HeLa cells. M, mock infected; I, RSV infected. (C) Streptavidin–HRP blot of immunoprecipitates from lysates shown in B, using antibodies against RSV G protein or caveolin-1. Representative data of two independent experiments are shown.

We performed IPs on non-crosslinked cell lysates from RSV-infected and mock-infected cells using anti-G, anti-M and anti-M2-1 antibodies. These lysates were prepared using milder detergent and salt concentrations. Interactions between the G, M and M2-1 proteins were identified (Fig. 6A), validating that viral protein–protein interactions were maintained under these extraction conditions. Importantly, caveolin-1 specifically co-immunoprecipitated with the viral G protein. Caveolin-1 was also detected in the M and M2-1 protein IPs, as expected, but in both cases the amount of caveolin-1 precipitated was lower compared to that in the G protein IP. This provided evidence for an interaction between caveolin-1 and the viral G and M protein complex in RSV-infected cells.

To test whether this interaction takes place on the surface of infected cells, we used a previously described surface biotinylation assay (Low et al., 2008). Mock-infected and RSV-infected cells were treated with Sulfo-NHS-LC-LC-biotin and lysates subjected to IPs with caveolin-1 or G protein antibodies (Fig. 6B,C). Blotting of the IPs using streptavidin–HRP revealed the presence of a biotinylated protein species of the expected size of the G protein in both the G protein IP and the caveolin-1 IP (Fig. 6C). We conclude that caveolin-1 interacts with the RSV G and M protein complex on the surface of virus-infected cells. We further suggest that this interaction takes place in the RSV envelope.

### Caveolae are not required for RSV morphogenesis

**Fig. 7.**
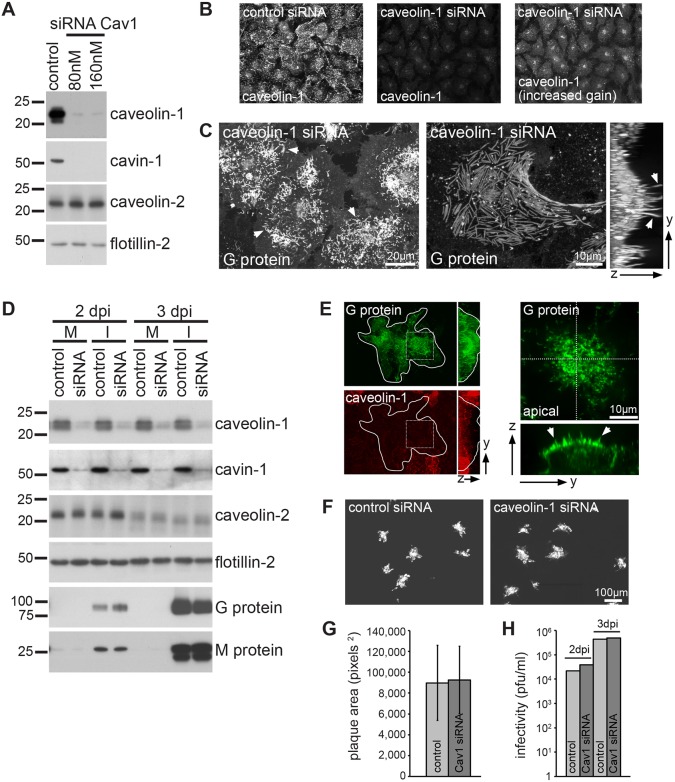
**Caveolae are not required for RSV filament morphogenesis.** (A) Immunoblots of HeLa cell lysates transfected with caveolin-1 siRNA or control siRNA. Lysates were prepared 4 days post transfection and probed with the indicated antibodies. Shown is a representative of five independent experiments. (B) Confocal micrographs of control and caveolin-1-siRNA-treated HeLa cells stained with antibodies against caveolin-1. (C) Maximum intensity projection (left) and 3D reconstruction of confocal stacks (right) of HeLa cells treated with 80 nM caveolin-1 siRNA for 3 days, infected with RSV (MOI 3) for 22 h, and fixed and stained with anti-G protein antibodies. Note the abundance of RSV filaments (arrowheads). (D) Immunoblots of HEp-2 cell lysates. Cells were transfected with 80 nM caveolin-1 siRNA or control siRNA for 3 days and either infected (I) with RSV (MOI 0.0001) or mock infected (M). Cell lysates were prepared at 2 dpi and 3 dpi, and probed with the indicated antibodies. (E) Representative micrographs of HEp-2 cells transfected with caveolin-1 siRNA and infected with RSV (MOI 0.0001). Cells were fixed 2 dpi and stained with anti-G and anti-caveolin-1 antibodies. The outline of the plaque is shown. Images on the right show the boxed region on the left. Note the presence of RSV filaments on the apical surface of the plaques (arrowheads). (F) Representative fluorescence micrographs of RSV-infected (MOI 0.0001) HEp-2 cells transfected with caveolin-1 siRNA or control siRNA. Cells were fixed 2 dpi and stained with anti-RSV antibody. (G) Quantification of plaque size at 2 dpi. Bar graphs show the mean plaque area, error bars indicate standard deviation (*n*=132 plaques each). (H) Virus titers determined at 2 dpi and 3 dpi. D–H show representative data from two independent experiments.

In a final analysis, we examined whether caveolae played a direct structural role in the assembly of the RSV envelope, i.e. if loss of caveolae prevented RSV biogenesis, transmission and infection. Caveolin-1 protein expression in HeLa and HEp-2 cells was downregulated by using specific siRNAs. Treatment with siRNA resulted in a near-complete loss of caveolin-1 and, as expected, a near-complete loss of cavin-1 protein expression (Fig. 7A,B,D). Although this would be expected to cause a profound reduction in the number of caveolae (Drab et al., 2001; Hill et al., 2008; Liu and Pilch, 2008; Liu et al., 2008), siRNA treatment failed to prevent virus filament formation and did not alter RSV protein expression (Fig. 7C,D). In addition, cell-to-cell virus transmission (Huong et al., 2016) and the production of infectious virus progeny were not notably impaired in cells lacking caveolae (Fig. 7E–H). These observations indicate that the recruitment of caveolae into RSV filaments is not essential for the formation of infectious virus.

## DISCUSSION

Although caveolin-1 was previously shown to be sequestered into RSV filaments during virus morphogenesis (Brown et al., 2002a,b; Kipper et al., 2015), it was unclear whether this association was dependent or independent of caveolae. In this study, we have shown that RSV morphogenesis occurs within caveolae, and that caveolae are actively recruited to and incorporated into the growing RSV envelope. We further demonstrate that RSV modifies and interacts with the caveolar coat machinery. We propose, therefore, that caveolae are hijacked during RSV assembly, and we hypothesize that the incorporation of caveolae into the virus contributes to the biophysical properties of the RSV envelope. This process may be a universal feature of different enveloped viruses that associate with caveolin-1.

**Fig. 8.**
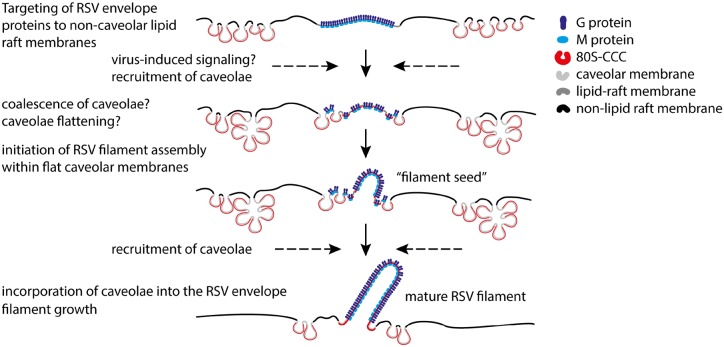
**Model of RSV envelope biogenesis.** RSV envelope proteins (only the RSV G and M proteins are shown for simplicity) are targeted to non-caveolar lipid raft membranes (Fleming et al., 2006; Brown et al., 2004; McCurdy and Graham, 2003). Caveolae are recruited to such membrane domains just prior to the initiation of RSV filament assembly. Virus-induced changes to the caveolar membrane coat (80S-CCC) and the caveolar lipid profile might induce clustering and coalescence of caveolae at the assembly site and flattening of the caveolar coat. This initiates RSV filament assembly within flat caveolar membranes. Caveolae are subsequently recruited to the growing filament and incorporated into the viral envelope.

We propose a model (Fig. 8) in which RSV envelope proteins (such as G and F proteins) are initially targeted to non-caveolar membrane domains that demarcate sites for viral filament assembly. Caveolae are recruited to these ‘filament precursors’ just prior to the initiation of RSV assembly. This process is dependent upon an intact actin network, and is likely to be triggered by virus-induced signaling and an interaction between caveolin-1 and components of the viral envelope. We demonstrate that caveolin-1 interacts with the G and M protein complex on the surface of infected cells, which is in line with previous reports showing that the RSV M and G proteins interact directly (Ghildyal et al., 2005; Henderson et al., 2002), and that the M protein interacts with caveolin-1 when both proteins are transiently overexpressed in non-infected 293T cells (Kipper et al., 2015). This association provides a potential mechanism for the recruitment of caveolae to sites of RSV assembly. Whether EHD2, an ATPase that links caveolae to the actin cytoskeleton to regulate caveolae dynamics (Stoeber et al., 2012), and other caveolar proteins such as cavin-2, cavin-3 and pacsin-2 (Hansen and Nichols, 2010; Shvets et al., 2014) are involved in the recruitment process remains to be tested. The subsequent induction of RSV filament growth within caveolar membranes is likely to be preceded by the coalescence of caveolae and the flattening of the caveolar membrane bulb. Three independent observations suggest that caveolae flattening might be caused by direct virus-induced changes to the caveolar protein coat. First, RSV infection caused a significant increase in cavin-1 protein levels, which coincided in time with the initiation of RSV assembly and with the incorporation of caveolae into nascent viral filaments. Second, upregulation of cavin-1 protein correlated with an increased number of cavin-1 molecules in the isolated caveolar coat complex, indicating that RSV directly alters the stoichiometry of the caveolar coat complex. Third, selective EM labeling of caveolin-1 showed that the 80S-CCC decorating the viral envelope produces a membrane coat distinct to that found in caveolar membranes. Although this remains speculative at present, the observed alteration to the stoichiometry of the 80S-CCC might cause a structural or conformational reorganization of the caveolar coat. This could potentially lead to caveolae flattening and the incorporation of the flattened caveolar protein coat and caveolar lipids into the growing viral envelope. RSV infection also alters the lipid composition of raft membranes (Yeo et al., 2009), and specific phospholipid-modifying enzymes have been proposed to control caveolae formation (Inaba et al., 2016). It is conceivable, therefore, that RSV-induced changes to the caveolar lipid profile contribute to the initiation of RSV filament assembly within caveolar membranes, and to their incorporation into the RSV envelope.

RSV morphogenesis and infection were not notably impaired in cultured cells lacking caveolae. Although our knockdown data agree with recent data by Kipper and colleagues (2015), the same authors observed a modest 2-fold reduction in RSV infectivity upon siRNA-mediated depletion of caveolin-2. Despite being a prominent component of caveolae, caveolin-2 is not required for caveolae formation (Razani et al., 2002), and depends on caveolin-1 for Golgi export and transport to the cell surface (Mora et al., 1999; Parolini et al., 1999). Thus, the observed 2-fold reduction in RSV infection upon caveolin-2 knockdown (Kipper et al., 2015) cannot be taken as evidence for a role of caveolae in RSV morphogenesis. Although a caveolae-independent function for caveolin-2 in RSV infection cannot be entirely ruled out (de Almeida et al., 2011; Razani et al., 2002), it is unclear whether a very moderate reduction in virus titer is consistent with an important role for caveolin-2 in RSV morphogenesis.

Previous reports have proposed a direct role of caveolin-1 in the morphogenesis and infection of influenza virus, PIV-5, and dengue virus (Ravid et al., 2010; Sun et al., 2010; García Cordero et al., 2014). This evidence is largely based on overexpression of caveolin-1 mutants (Ravid et al., 2010; Sun et al., 2010) and the use of complementary cell lines lacking or overexpressing wild-type caveolin-1 (Ravid et al., 2010). The caveolin-1 mutants P132L and F92A/V94A were originally proposed to behave in a dominant-negative fashion (Lee et al., 2002; Nystrom et al., 1999), and thus have been widely used to block caveolae formation. However, more recent studies suggest that these mutants do actually not affect the biogenesis of caveolae, and so do not act in a dominant-negative manner (Ren et al., 2004; Shatz et al., 2010). Overexpression of wild-type caveolin-1 (in particular in cell types that do not express the full complement of proteins required for making caveolae) is problematic too, as it can lead to the artefactual presence of caveolin-1 in non-caveolar membranes and to missorting of the protein into late endosomes and lysosomes (Hanson et al., 2013; Hayer et al., 2010b). Thus, viral phenotypes observed upon overexpression of caveolin-1 constructs should be interpreted with caution. Even if one assumes that caveolin-1 mutants do directly and specifically interfere with caveolae formation, the moderate reduction in virus infectivity observed in cells that are proposed to express non-functional caveolin-1 (Sun et al., 2010; Ravid et al., 2010) does not suggest a direct structural role for caveolae in these enveloped viruses.

We propose that caveolae play a post-assembly role in RSV morphogenesis and infection. There is evidence from work on other viruses that the incorporation of lipid raft components into the viral envelope is critical for specific post-assembly stages of virus infection. For example, the HIV-1 envelope has a lipid composition similar to that expected in lipid rafts (Brugger et al., 2006; Aloia et al., 1993), and the presence of cholesterol in the viral envelope has been shown to mediate efficient membrane fusion during virus entry (Campbell et al., 2004). Entry of influenza virus appears to rely on a similar mechanism (Sun and Whittaker, 2003). Interestingly, the lipid compositions of caveolar membranes and the RSV envelope are also strikingly similar; both membrane structures are rich in cholesterol, PS and PI(4,5)P_2_ (Fujita et al., 2009; Zhang et al., 2009; Yeo et al., 2009). Caveolin-1 has been reported to bind cholesterol (Murata et al., 1995), and there is ample evidence that caveolae control the lipid composition of cellular membranes (Pilch and Liu, 2011; Ariotti et al., 2014), as well as the trafficking of cholesterol and glycosphingolipid within the cell (Pol et al., 2001; Shvets et al., 2015; Sharma et al., 2004; Cheng et al., 2006). Furthermore, caveolae are an extremely abundant feature of the plasma membrane in many cell types (Stan, 2005), and so are ideally suited to serve as a storage reservoir and trafficking device for particular membrane lipids. It is tempting to hypothesize, therefore, that RSV exploits caveolae as a source for cholesterol and other raft lipids, which in the absence of caveolae, are provided by alternative and as yet unidentified lipid raft membranes. A second possibility is that caveolae are involved in the delivery of certain host cell proteins into the RSV envelope and that these proteins are largely dispensable for virus assembly, replication and infection in cell culture. There is precedent for this notion. For example, RSV lacking the small hydrophobic (SH) membrane protein replicate normally in cultured cells, but are attenuated in a mouse model (Russell et al., 2015). In addition, complement control proteins such as CD55 and CD59 have been identified in RSV virions (Brown et al., 2004), and there is evidence from work on other viruses that the incorporation of such proteins into the viral envelope provide protection against complement attack (Spear et al., 1995; Amet et al., 2012; Ejaz et al., 2012; Montefiori et al., 1994; Rangaswamy et al., 2016; Saifuddin et al., 1997). Viruses lacking such molecules would be infectious in cell culture, but would exhibit impaired infectivity *in vivo*. Since CD59 and other immune-modulators are present in detergent-resistant membrane fractions enriched for caveolar components (Sprenger et al., 2006) and glycosylphosphatidylinositol (GPI)-anchored proteins can become concentrated within caveolae (Mayor et al., 1994), it is possible that caveolae are involved in the transport of such proteins into the viral envelope. Finally, the role of caveolae in RSV morphogenesis and infection might be specific to the cell type. Interestingly, RSV infection occurs in ciliated airway epithelial cells (Jumat et al., 2015), in which caveolae are abundant (Krasteva et al., 2006). Therefore, caveolae might play a much more prominent role in RSV transmission and infection in the airway epithelium where RSV infection naturally occurs.

In conclusion, our data provide strong evidence that RSV utilizes caveolae during viral filament assembly. Contrary to previous suggestions, we propose that caveolae play a post-assembly role rather than a direct structural role in virus morphogenesis. Such putative functions for caveolae in the RSV life cycle cannot be readily addressed in cell culture models and may not lead to a detectable phenotype in virus particle formation. Specific assays are required that allow these questions to be addressed in the context of a whole-animal system.

## MATERIALS AND METHODS

### Antibodies, cell lines and cell culture

The following antibodies were used: mouse anti-GFP [1:2000 for western blotting (WB); Roche, Mannheim, 11814460001], rabbit anti-PTRF (cavin-1) [1:2000 for WB, 1:200 for immunofluorescence (IF); Abcam, Cambridge, ab48824], rabbit anti-caveolin-1 (1:10,000 for WB, 1:500 for IF; BD Biosciences, 610060), mouse anti-caveolin-2 (1:1000 for WB; BD Biosciences 610685), mouse anti-flotillin-2 (1:5000 for WB, 1:500 for IF; BD Biosciences, 610383), mouse anti-G protein (1:500 for IF, 1:2000 for WB; Abcam, Cambridge, mab8582), rabbit anti-F protein (1:200 for IF), mouse anti-RSV (1:500 for IF, NCL-RSV3, Novocastra Laboratories) and mouse anti-filaminA (1:2000 for WB; Santa Cruz Biotechnology, sc-17749). The following mouse monoclonal antibodies were described previously (Brown et al., 2004): mouse anti-M (1:1000 for WB), mouse anti-M2-1 (1:1000 for WB). Streptavidin conjugated to horseradish peroxidase (HRP), CellMask™Orange, phalloidin–FITC, and Alexa Fluor 488, 555, 633- and HRP-conjugated secondary antibodies were from LifeTechnology, Singapore. Cycloheximide was from Sigma, Singapore. The clonal HeLa cell line stably expressing cavin1–EGFP has been described previously (Ludwig et al., 2013). HeLa and Hep-2 cells were cultured in Dulbecco's Modified Eagle Medium (DMEM), 10% fetal calf serum (FCS), penicillin-streptomycin (LifeTechnology, Singapore) at 37°C and under a 5% CO_2_ atmosphere.

### RSV infection

HeLa or HEp-2 cells were infected with RSV at a multiplicity of infection (MOI) 3 (normal MOI) or 0.0001 (low MOI). During infection, cells were grown in DMEM, 2% FCS and penicillin-streptomycin at 33°C and under a 5% CO_2_ atmosphere. Mock-infected cells were treated in the same way except that no virus was added. RSV particles were isolated as described previously (Radhakrishnan et al., 2010).

### Indirect immunofluorescence and confocal microscopy

For indirect immunofluorescence, cells were fixed in 4% paraformaldehyde in PBS pH 7.4 for 10 min or in methanol at −20°C for 10 min. Cells were incubated with primary antibody in PBS, pH 7.4, 5% FCS and 0.2% saponin for 2 h or overnight. Secondary antibodies were used in the same buffer for 1 h. Confocal microscopy was carried out on a CorrSight confocal spinning disk microscope (FEI Company) equipped with an Orca R2 CCD camera (Hamamatsu) using a 40× oil objective (NA 1.3, EC Plan Neofluar M27, Zeiss) or a 63× oil objective (NA 1.4, Plan Apochromat M27, Zeiss) and standard filter sets. All multi-color micrographs were acquired with the 63× objective. Images were processed in ImageJ or Fiji (Schindelin et al., 2012; Schneider et al., 2012) and Photoshop CS6.

### Live-cell imaging

HeLa cells stably transfected with cavin1–EGFP and infected with RSV (MOI 3) were grown on fibronectin-coated glass-bottom dishes (MatTec Corp., Ashland, MA) and imaged live in DMEM without Phenol Red supplemented with 2% FCS on the CorrSight confocal spinning disk microscope (FEI Company). Imaging was carried out at 33°C, 5% CO_2_ and 90% humidity in a closed atmosphere chamber (IBIDI). Up to 20 different locations were imaged simultaneously using the multi-stage position function in LA software (FEI Company). Confocal images were acquired with a 40× oil objective (NA 1.3, EC Plan Neofluar M27, Zeiss) using the 488 nm laser line (65 mW; iChrome MLE-LFA) and a standard GFP filter set. Focus was maintained by a hardware autofocus system (Focus Clamp). The laser output power and exposure times were set to a minimum. Time-lapse recordings were analyzed in ImageJ or Fiji (Schindelin et al., 2012; Schneider et al., 2012).

### Surface biotinylation and immunoprecipitation

This was performed as described previously (Low et al., 2008). Briefly HEp-2 cells were infected with RSV at an MOI of 3 for 20 h. Surface biotinylation was carried out with 0.5 mg/ml EZ-Link Sulfo-NHS-LC-LC-Biotin (Pierce Biotechnology) solution in PBS pH 8 for 1 h at room temperature. Cells were lysed in ice-cold RIPA buffer (PBS pH 8, 1% NP-40, 1 mM EDTA, 0.1% SDS and 2 mM PMSF) at 4°C for 20 min. Cleared lysates were incubated with antibodies overnight at 4°C in binding buffer [PBS pH 8, 0.5% NP-40, 1 mM EDTA, 0.25% BSA (w/v) and 2 mM lysine]. Protein-A–Sepharose (Sigma) was added into the mixture and incubated for 90 min under slow rotation. Pellets were washed thrice in PBS pH 8, 1 mM EDTA and 1% Triton X-100. Bound proteins were eluted by addition of SDS-PAGE sample buffer.

### DSP crosslinking, sucrose density gradients and immunoprecipitation of the 80S-CCC

Confluent cultures of HeLa cells were mock-infected or RSV-infected (MOI 3) and crosslinked at 22 hpi with 2 mM dithiobis(succinimidyl propionate) (DSP; LifeTechnology, Singapore) as described previously (Ludwig et al., 2013). Cells were scraped into lysis buffer (LB) [50 mM Tris-HCl pH 8, 300 mM NaCl, 5 mM EDTA, 1% (v/v) Triton X-100, 1% (w/v) octyl-glucoside, and protease inhibitor cocktail (Roche, Mannheim)] and cleared by centrifugation (20,000 ***g*** for 30 min). Lysates were loaded on top of a linear 10–40% (w/v) sucrose gradient prepared in 50 mM Tris-HCl pH 8, 300 mM NaCl and 0.2% Triton X-100. Gradients were spun in a SW41Ti rotor at 37,000 rpm for 6 h at 4°C. 12 1 ml fractions were collected from the top of the gradient. For immunoprecipitations, the peak fractions were pooled and 1 ml of each incubated with 30 µl of GFP-trap or RFP-trap resin (Chromotec) overnight. Beads were washed four times with 1 ml of 50 mM Tris-HCl pH 8, 300 mM NaCl and 0.2% Triton X-100 and once with Tris-HCl pH 8 and 150 mM NaCl. Bound proteins were eluted by addition of 2× SDS-PAGE sample buffer.

### Electron microscopy and 3D tomography

HeLa cells were grown on fibronectin-coated glass-bottom dishes (MatTec Corp., Ashland, MA) and transfected with 1 µg caveolin-1–APEX2–EGFP plasmid DNA using FugeneHD (Promega, Singapore). At 18–24 h post transfection, cells were infected with RSV (10 MOI) and fixed after 20–24 hpi with 2% glutaraldehyde (EMS, Hatfield, PA) and 2 mM CaCl_2_ in 0.1 M cacodylate buffer pH 7.4 (CB) (EMS) for 1 h on ice. APEX labeling was carried out as described previously (Ludwig et al., 2013, 2016). Electron micrographs were recorded on a Tecnai T12 (FEI Company) TEM operated at 120 kV using a 4k×4k Eagle (FEI) CCD camera. Single-axis tilt series were recorded at ±64°, recording an image at 2° intervals. The nominal magnification was 18,500×, corresponding to an object pixel size of 5.9 Å. Tilt series were binned by a factor of two and reconstructed by filtered back-projection with the IMOD software package (Kremer et al., 1996).

### siRNA transfection

On-target Plus SMART pool siRNAs against human caveolin-1 and non-targeting control siRNAs were from Thermo Scientific. HeLa or HEp-2 cells were transfected at 30% confluency using Oligofectamine (LifeTechnology, Singapore) and 80 nM siRNA according to the manufacturer's instructions. Cells were reseeded onto fibronectin-coated coverslips at 3 days post transfection, and infected with RSV (MOI 3 for HeLa and MOI 0.0001 for HEp-2) at 4 days post transfection in DMEM without serum.
